# The versatility and paradox of BMP signaling in endothelial cell behaviors and blood vessel function

**DOI:** 10.1007/s00018-021-04033-z

**Published:** 2022-01-19

**Authors:** Molly R. Kulikauskas, Shaka X, Victoria L. Bautch

**Affiliations:** 1 Curriculum in Cell Biology and Physiology, The University of North Carolina at Chapel Hill, Chapel Hill, NC 27599 USA; 2 Department of Biology, The University of North Carolina at Chapel Hill, Chapel Hill, NC 27599 USA; 3 Present Address: Department of Biomedical Engineering, Yale University, New Haven, CT USA; 4 McAllister Heart Institute, The University of North Carolina at Chapel Hill, Chapel Hill, NC 27599 USA

**Keywords:** BMP, TGFβ, Angiogenesis, Vascular homeostasis, Vascular disease, Adherens junctions

## Abstract

Blood vessels expand via sprouting angiogenesis, and this process involves numerous endothelial cell behaviors, such as collective migration, proliferation, cell–cell junction rearrangements, and anastomosis and lumen formation. Subsequently, blood vessels remodel to form a hierarchical network that circulates blood and delivers oxygen and nutrients to tissue. During this time, endothelial cells become quiescent and form a barrier between blood and tissues that regulates transport of liquids and solutes. Bone morphogenetic protein (BMP) signaling regulates both proangiogenic and homeostatic endothelial cell behaviors as blood vessels form and mature. Almost 30 years ago, human pedigrees linked BMP signaling to diseases associated with blood vessel hemorrhage and shunts, and recent work greatly expanded our knowledge of the players and the effects of vascular BMP signaling. Despite these gains, there remain paradoxes and questions, especially with respect to how and where the different and opposing BMP signaling outputs are regulated. This review examines endothelial cell BMP signaling in vitro and in vivo and discusses the paradox of BMP signals that both destabilize and stabilize endothelial cell behaviors.

## Introduction and historical links

Blood vessels form during embryonic development in response to signals that originate from developing tissues and organs, and many of these same pathways remain engaged as blood vessels remodel and transition to homeostasis. Among these signaling pathways are some dedicated to vessel formation and maintenance, while other pathways are also utilized in multiple developmental programs. BMP (bone morphogenetic protein) is an example of a signal that is used iteratively throughout metazoan development. Our understanding of how BMP signaling affects vascular development and function initially lagged behind that of signals more dedicated to blood vessel functions such as VEGFA, as rigorous examination of BMP pathway function in vivo required the ability to manipulate signaling both spatially and temporally. An understanding of vascular BMP signaling has proven particularly elusive, as numerous pathway components show complex and context-dependent vascular phenotypes when manipulated. For example, global genetic deletion of BMP/TGFβ pathway components in mice yielded complex phenotypes, suggesting an important role for the TGFβ superfamily (that includes BMP) in cardiovascular development, but early lethality and the co-occurrence of both cardiac and vascular defects made these phenotypes difficult to interpret [1]. These complex in vivo phenotypes in turn make interpretation of in vitro outputs with cultured endothelial cells challenging and have stymied the generation of unifying principles. However, clear links between human cardiovascular disease and BMP signaling compel further understanding of vascular BMP signaling, and recently several concepts regarding BMP pathway function in the vasculature have emerged.

The BMP pathway is part of the larger transforming growth factor β (TGF β) pathway family, first identified as regulators of wing patterning in *Drosophila* via invertebrate Decapentaplegic (DPP) [2, 3]. BMP provides ventralizing cues that contribute to setting up the dorsal–ventral body axis prior to and during gastrulation, and loss of BMP signaling at this stage dorsalizes embryos, preventing further development [4]. BMP also regulates aspects of bone differentiation later in development, and differentiation of several cell types from stem cells [5, 6]. These non-vascular studies indicate that BMP signaling is used in diverse developmental processes. Additionally, BMP signaling often integrates with other signaling pathways, such as VEGFA, Wnt, FGF, and Notch, in complex patterns of pathway crosstalk.

Evidence that BMP signaling regulates vascular development and function initially came from two lines of experimental evidence. First, cell-based studies showed that endothelial cells migrate and proliferate in response to BMP signals [7]. These early studies were only possible once conditions for the culture and propagation of endothelial cells in vitro were established by Gimbrone and colleagues [8]. Second, early analysis of human families where a vascular disease called Hereditary Hemorrhagic Telangiectasia (HHT) segregated showed clear linkage to the BMP pathway genes ALK1 (HHT2) and endoglin (ENG, HHT1) [9, 10]. This disease is characterized by several vascular defects, including hemorrhage, nose-bleeds, and arteriovenous shunts or malformations (AVMs). Further analysis of human genetic data linked another pathway component, SMAD4, to HHT [11], and numerous mouse genetic models of gene disruption recapitulate aspects of the human defects (see below), firmly linking BMP signaling to vascular function. Nevertheless, a unified model describing BMP function in blood vessels is lacking, and mounting evidence indicates that BMP signaling has versatile and often opposing outcomes in the vasculature that depend on numerous variables both intrinsic and extrinsic to the pathway. For example, in some contexts, BMP signaling is proangiogenic and promotes blood vessel sprouting, while in other contexts, BMP signaling is homeostatic, and promotes blood vessel quiescence. Although BMP signals affect several vascular cell types, the nexus of BMP signaling integration for proangiogenic vs. homeostatic outputs are the endothelial cells that line all blood vessels and regulate vessel network expansion during development and barrier function in adults.

This review will describe the BMP pathway components most relevant to blood vessel development and function (for more comprehensive pathway reviews see [12–15]). Because the endothelial cell is the first and often primary cell type in the vascular response to BMP signaling inputs, this review will focus on BMP signaling effects on endothelial cells. The effects of TGFβ/BMP signaling on non-endothelial vascular cells such as smooth muscle cells are discussed in [16, 17]. Although canonical TGFβ signaling is important for endothelial cell behaviors [18], here we focus on signaling initiated by BMP ligands and transduced by BMP receptor complexes, since much of the versatility in vascular responses results from the differential responses of endothelial cells to BMP signaling inputs. Finally, BMP signaling activates both canonical pathways (described below) that result in nuclear translocation of phosphorylated effectors called SMADs and changes in gene transcription, and non-canonical pathways that signal independent of SMAD effectors. In this review, we primarily focus on the effects of canonical BMP signaling, which are strongly associated with vascular phenotypes.

## Chapter 1: BMP signaling in blood vessels—the players

### Overview

**Fig. 1:**
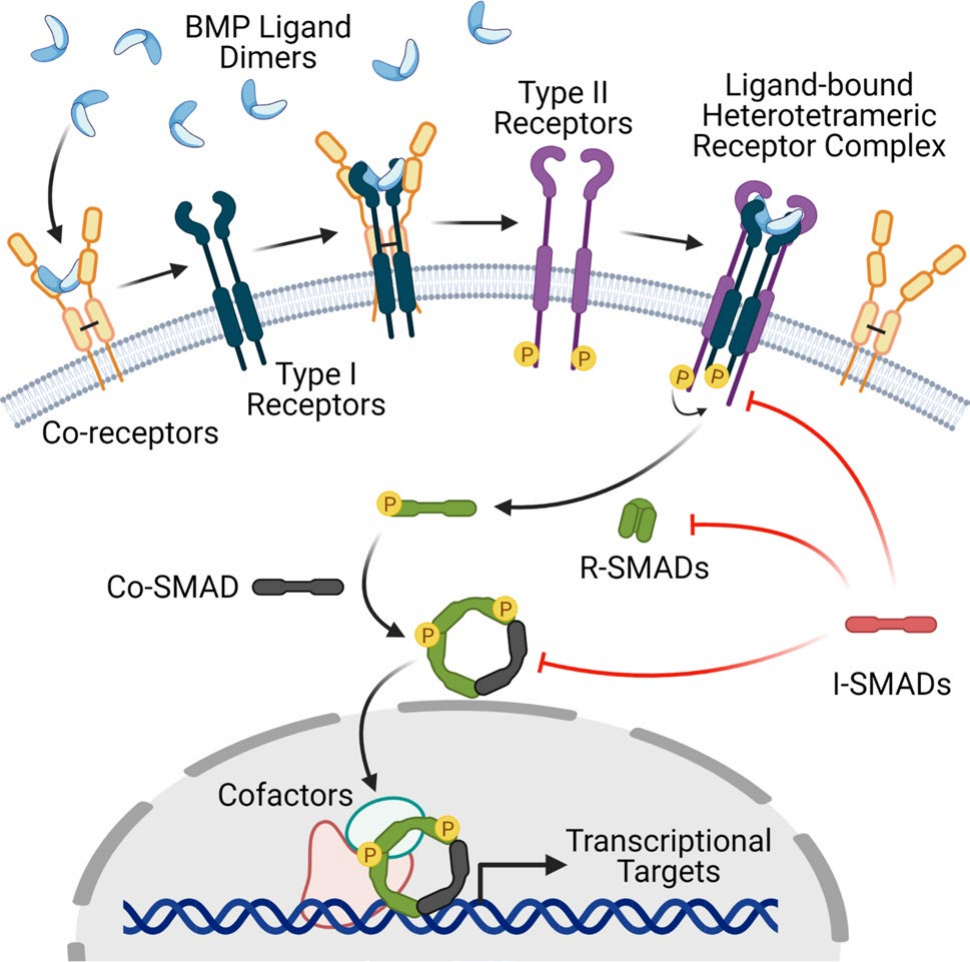
Overview of the Canonical BMP Signaling Pathway. BMP ligand dimers bind Type I receptors with the help of Co-receptors. The ligand-bound Type I receptors form a heterotetrameric receptor complex with Type II receptors. The Type II receptor phosphorylates and activates the Type I receptor, which then phosphorylates the R-SMAD and changes it to an active conformation that complexes with the Co-SMAD and translocates into the nucleus to regulate transcription. i-SMADs negatively regulate this pathway at several points

Canonical BMP signaling occurs via a core set of molecular events in all cell types (Fig. 1) (for in-depth reviews of vascular BMP and TGFβ signaling, see [1, 19–22]). Briefly, secreted BMP ligands form dimers that bind to heterotetrameric receptor complexes in the cell membrane; these receptor complexes contain both Type I and Type II serine–threonine kinase BMP receptors. Upon ligand binding, Type II receptors phosphorylate and activate Type I receptors in the same complex, then Type I receptors phosphorylate cytoplasmic proteins called Receptor-mediated SMADs (R-SMADs, SMAD1/5/8) that interact with the intracellular domain of the Type I receptor. Phosphorylated R-SMADs are released from the receptor complex and next bind to the Common SMAD (Co-SMAD, SMAD4) in the cytoplasm. SMAD4 acts as a chaperone to translocate the R-SMAD/Co-SMAD complexes to the nucleus, where they transcriptionally regulate target genes.

Co-receptors (sometimes called Type III receptors), such as betaglycan and endoglin (ENG), are membrane-localized BMP pathway components that lack intrinsic kinase activity but instead enhance signaling via receptor interactions. Two inhibitory SMADs (i-SMADs), SMAD6 and SMAD7, negatively regulate BMP signaling in a cell-intrinsic manner, likely through interactions with Type I receptors and the R-SMADs. BMPER (BMP endothelial cell precursor-derived regulator, also called Crossveinless-2) is another cell-intrinsic BMP pathway regulator that acts both negatively and positively on BMP signaling, depending on the context. Other negative regulators of BMP signaling are primarily cell-extrinsic, including Noggin, Chordin, Follistatin, and Gremlin; these antagonists primarily bind BMP ligands in the extracellular space, preventing their binding to receptors.

BMP signaling is complex, as several different ligands, receptors, and co-receptors are often involved, even in a single cell type. Although the binding preferences of different BMP ligands for receptor complexes are thought to primarily result from differences in the Type I receptors, these data are largely derived from biochemical experiments in vitro, and how these preferences translate to in vivo situations is unclear. It is also not well-understood how co-receptors influence ligand binding to surface receptors, how competition among different BMP receptor complexes plays out in cells, and how ligands are presented to receptors (i.e., homodimers vs. heterodimers). This variety and complexity likely contribute to the disparate cellular phenotypes that confound generation of simple molecular models of BMP function. BMP signaling complexity is even more relevant in the endothelial cells of blood vessels, where canonical BMP signaling as described above leads to opposite cellular phenotypes—either proangiogenic or homeostatic (anti-angiogenic) cell behaviors, in ways that are poorly understood. Below we describe the BMP pathway components thought to be most relevant to BMP signaling in endothelial cells.

### BMP ligands involved in endothelial cell function

**Fig. 2:**
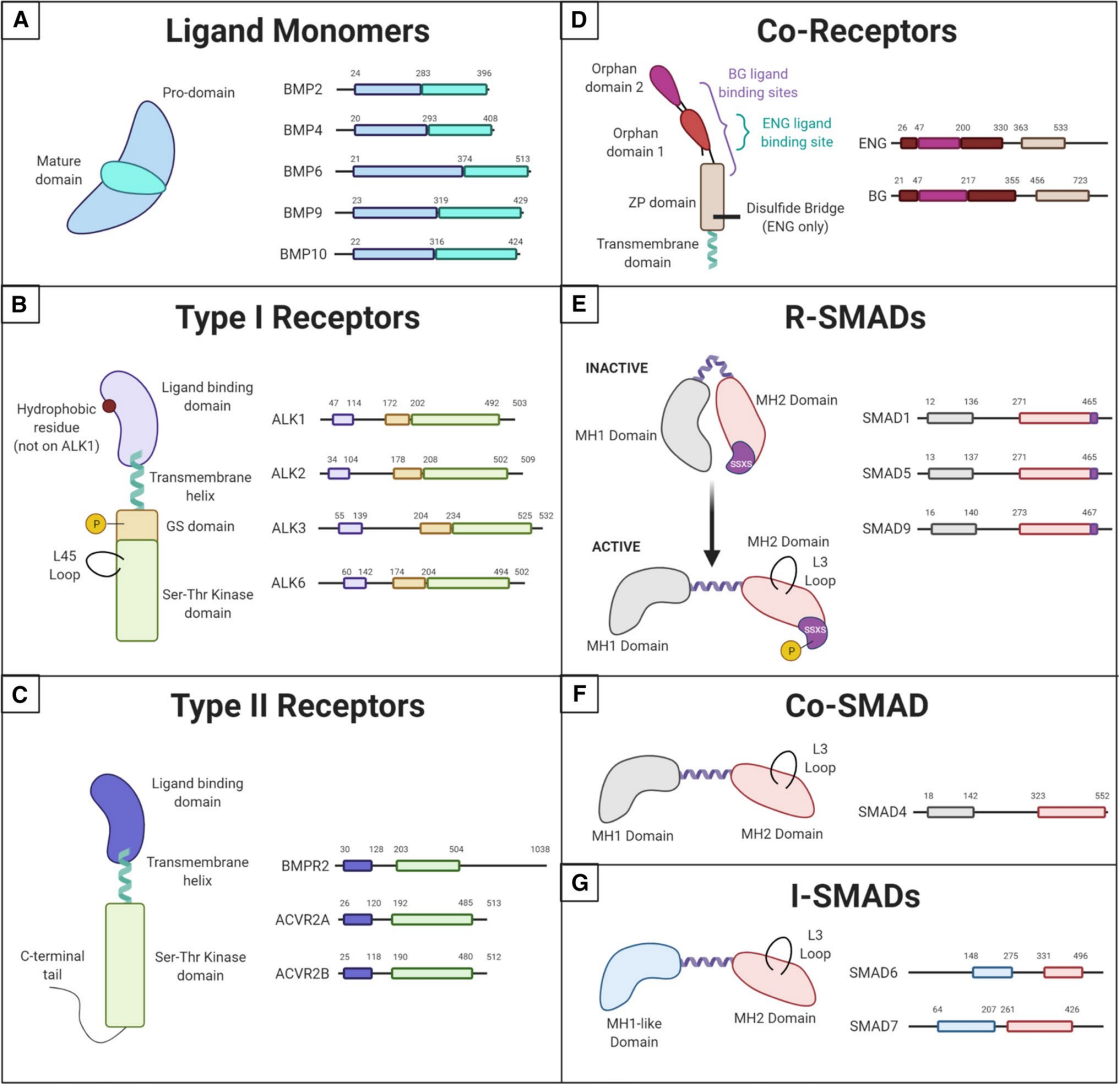
Structure of BMP Pathway Components. Comparison of the structures of different components used in endothelial cell BMP signaling, highlighting important functional domains. **A** BMP ligand monomers have a pro-domain and a mature domain. Ligands often pair to make homodimers, although heterodimers have been reported. **B** Type I receptors share a ligand-binding domain with a hydrophobic residue on ALK2, 3, and 6, but not ALK1. They are phosphorylated by Type II receptors on their GS domain. Their active site is in the Ser–Thr kinase domain on the C-terminal end of the protein. This domain also contains the L45 loop that binds the R-SMAD L3 loop. **C** Type II receptors have a similar structure to Type I receptors but lack the GS domain and some contain a long C-terminal tail important for non-canonical signaling. **D** BMP Co-Receptor endoglin (ENG) dimerizes through a disulfide bridge in its ZP domain and binds ligands in the Orphan Domain 1. Betaglycan (BG) is a monomer that wraps around its ligand with both Orphan Domains and the N-terminal region of its ZP domain. Both co-receptors have short transmembrane domains with no signaling functionality. **E**–**G** All SMADs contain an MH2 domain with an L3 loop capable of binding the L45 loop on Type I receptors, connected to an MH1 domain (or MH1-like domain in i-SMADs) by a linker region. R-SMADs maintain an inactive, folded conformation until they are phosphorylated on the SSXS motif within their MH2 domain by Type I receptors

Numerous BMP ligands share a general structure (Fig. 2A) but differ in their receptor-binding preferences [23]). Here we focus on BMP2, BMP4, BMP6, BMP9, and BMP10, as they interact most strongly with Type I receptors that initiate signaling in endothelial cells. Various vascular developmental defects were found in mouse and zebrafish embryos lacking *Bmp9* and/or *Bmp10* [24–30]; however, the roles of BMP4 and BMP6 in embryonic vascular development are less clear, as mice lacking *Bmp4* failed to differentiate mesoderm and died shortly after gastrulation, while mice globally lacking *Bmp6* were viable with mild metabolic abnormalities [31–33].

BMP ligands primarily signal to endothelial cells in a paracrine manner, although some ligands circulate at physiological levels in the bloodstream, while others are expressed by endothelial cells and may provide autocrine signaling [34]. BMP ligands regulate signaling by their concentration, bioavailability, and activity. Local availability of some BMP ligands to endothelial cells in vivo, such as BMP2, BMP4 and BMP6, is restricted by binding to the extracellular matrix, which limits diffusion and blocks access to receptors [35], and by interactions with secreted antagonists [36]. Although many BMP ligands are detected in serum, only BMP9 and BMP10 were found at levels suggesting a primary endocrine route to endothelial cells via the bloodstream. BMP10 is produced early in mouse development (E8.75) by cardiomyocytes, and in humans, adult expression is restricted to the heart with lower levels in the liver [37–39], while BMP9 is produced by the liver in humans and animal models [30, 39–41]. Both BMP9 and BMP10 have relatively high circulating levels in blood (~ 0.5–15 ng/mL) in both humans and mice [24, 28, 41–45], indicating that they are delivered to endothelial cells via the bloodstream. BMP2, BMP4, and BMP6 serum levels are much lower (pg/mL–low ng/mL range) [43, 46, 47] and are expressed in a temporally and spatially regulated manner developmentally [32, 48], indicating paracrine interactions that affect endothelial cell behaviors. Interestingly, BMP2 and BMP4 mRNA and protein are also expressed in endothelial cells, suggesting autocrine or paracrine vascular effects that have not been well-explored [49–55]. Although most work has characterized BMP ligand homodimers that initiate signaling, heterodimers also exist and may be relevant in some places; for example, BMP9/BMP10 heterodimers that signal through ALK1 are found in mouse and human blood and may be the main entity activating ALK1 signaling in vitro [39, 44].

### Endothelial cell BMP type I receptors

Type I BMP receptors are membrane-localized serine/threonine kinases that share significant overall homology. However, these receptors diverge substantially in their ligand-binding domains, leading to different affinities for BMP ligands and potentially different signaling outcomes. Type I receptors have non-redundant functions in endothelial cells, as evidenced by in vitro studies and the different vascular phenotypes of mouse loss-of-function mutations for the Type I receptors (see Table 1) [56]. It is likely that, along with ligand availability, differential expression of the Type I receptors is a major component of the heterogeneous responses of endothelial cells to BMP signaling inputs. The Type I BMP receptors most important for endothelial cell signaling are ALK1 (ACVRL1), ALK2 (ACVR1), and ALK3 (BMPRIA), based on both their expression in endothelial cells and the vascular defects that occur upon deletion in animal models [34] (see Table 1). ALK1 is the most highly expressed endothelial cell Type 1 receptor in vitro [57] and is broadly expressed in endothelial cells throughout mouse development; endothelial cell ALK1 expression is diminished and becomes largely restricted to the lungs in adult mice [54, 58]. In human primary endothelial cells, ALK3 is expressed at significantly lower levels than ALK1 and ALK2 [52, 53, 57]. Single cell RNA sequencing of adult mouse endothelial cells from different vascular beds detected the highest ALK3 levels in coronary vessels, with low to undetectable levels elsewhere [54]. ALK2 is broadly expressed in human endothelial cells, and single cell RNA sequencing displayed low but widespread expression of ALK2 in both arterial and venous endothelial cells from different tissues of adult mice [54, 59]. ALK6 (BMPR1B) is also expressed in endothelial cells, but levels are low and there is no obvious loss-of-function vascular phenotype [60, 61]. Type I receptors consist of an extracellular ligand-binding domain, a membrane-spanning domain, a glycine/serine-rich (GS) domain, and a serine–threonine kinase domain (Fig. 2B). The GS domain is the site of phosphorylation by Type II receptors, activating the kinase domain that is functionally responsible for phosphorylating R-SMADs.

**Table 1:** BMP pathway gene deletion: animal model phenotypes

Gene	Global deletion phenotype* (mouse except where stated zebrafish)	Endothelial cell-selective deletion phenotype (mouse except where stated zebrafish)
*Bmp2*	Embryo: Lethal (E7.5–10.5); Failed closure of pro-amniotic canal; malformed amnion/chorion, cardiac development defects [232]	Adult (Tie2-Cre): Hemochromatosis (serum and tissue iron overload), ↓ spleen iron [33, 233] Adult (Cdh5-Cre): Viable; no vascular phenotype reported [234, 235]
*Bmp4*	Embryo: Lethal (E6.5-E9.5); Defective mesoderm differentiation and blood island formation [31]	Adult (Cdh5-CreERT2) (Excised at 6–8weeks and challenged with thioglycolate): Diminished leukocyte infiltration in acute inflammation [146]
*Bmp6*	Embryo: (Late gestation) ossification delay [236] Adult: Viable, fertile, no overt defects, normal ossification in pups/adults [236] Adult: Hemochromatosis (serum and tissue iron overload), ↓spleen iron [32, 33]	Adult (Tie2-Cre): Hemochromatosis (serum and tissue iron overload), ↓ spleen iron [32, 33]
*Bmp9*	Late Embryo/Neonate: Disrupted lymphatic development (↑ LEC proliferation/enlarged lymph vessels, ↓ lymphatic valves) [25, 26] Post-Natal Retina: Viable; Normal vascular development. With BMP10 neutralizing antibody: ↑ vascular density and ↓ vascular expansion [24, 28] Zebrafish embryo (morpholino): Venous remodeling defects [27] Zebrafish embryo (mutant): Viable; no overt phenotype [30]	N/A
*Bmp10*	Embryo: Lethal (E10.0–10.5); ↓ cardiomyocyte proliferation (arrested cardiogenesis), ventricular hypoplasia, ↓ ventricular trabeculae (impaired trabeculation), abnormal endocardial cushion development, severely impaired cardiac function and circulation, vascular impairment [37]. Developmental arrest at E10.5, enlarged pericardium, AVM (DA and CV dilated and fused into single continuous channel), yolk sac: no vitelline vessels and stalled primary capillary plexus development [28] Zebrafish embryo (morpholino *bmp10* + *bmp10-like* double KD): AVMs in midbrain and hindbrain; enlarged cranial basal communicating artery [29] Zebrafish embryo (mutant, *bmp10* + *bmp10-like* double KO): Lethal high-flow cranial AVMs [30] Zebrafish juvenile/adult (mutant *bmp10*): Premature death, abdominal edema, enlarged/hemorrhagic skin blood vessels, disorganized liver vasculature, high-output heart failure [30]	N/A
*Bmpr2*	Embryo: Lethal (< E9.5); Pre-gastrulation developmental arrest, lack primitive streak and mesoderm [87] Embryo: (Mox2-Cre) Gastrulation and mesoderm defects, cardiac defects including double-outlet right ventricle (DORV), ventricular septal defect (VSD), AV cushion defects, thickened valve leaflets [237] Adult (*Bmpr2*^+/−^): ↑ mean arterial pressure and pulmonary vascular resistance; thickened muscularized pulmonary artery walls; ↑ alveolar-capillary units; mild pulmonary hypertension; impaired pulmonary vascular remodeling [171] Adult (*Bmpr2*^+/−^*;ApoE*^*−/−*)^: Accelerated atherosclerosis and ↑ endothelial inflammation in arteries [221]	Embryo (Tie2-Cre): AV cushion defects (atrial septal defect, membranous VSD, thickened valve leaflets) [237] Neonate (Tie2-Cre): Lethal (~ P7); Abnormal AV cushion remodeling, thickened semilunar valve formation [237] Post-Natal Retina (Cdh5-CreERT2): ↓ radial expansion, ↓ vascular density, and ↓ sprouting at angiogenic front [59] Adult (Alk1-Cre-L1, pulmonary EC): Predisposition to develop PAH (elevated right ventricular systolic pressure (RVSP)) associated with right ventricular hypertrophy and ↑ number and wall thickness of distal pulmonary arteries [172]. ↑ leaky pulmonary vessels, ↑ leukocyte infiltration into lungs [173] Adult (Tie2-rtTA x TetO_7_-Bmpr2^delx4^): ↑ RVSP; muscularization of small vessels; thrombosis, ↑ inflammatory cells, ↑ proliferating cells, moderate ↑ in apoptotic cells [238] Adult (Scl-CreERT, general EC): RVSP under hypoxic conditions (measure of PAH) [83]
*Alk1*	Embryo: Lethal (E10.5); Excessive capillary plexus fusion; impaired yolk-sac/embryonic vascular development; large vessel dilation; VSMC differentiation and recruitment defects [218]. AVMs between DA and CV by E8.5, and in multiple areas by E9.5 [191] Post-Natal Retina (Rosa26-CreER): (Excised at P3) Extensive, fully dilated AVMs at P5 [184] Neonate (Rosa26-CreERT2): ↑ density, ↑ # filopodia, and ↑ diameter of lymphatic vessels of various tissues. Blood vessels not assessed [26] Adult (Rosa26-CreER): Sex-dependent lethality 9–21 days post-excision. ↓ weight and hemoglobin levels, ↑ hemorrhage and anemia, enlarged heart, dilated pulmonary arteries and veins, AVMs in gastrointestinal tract, uterus, and wounded skin [184, 219] Zebrafish embryo (mutant *alk1*^*y6*^): Dilated high-flow cranial AVMs, ↑ number of endothelial cells in cranial vessels due to directed arterial EC migration [193]. Edema in head, pericardium, and yolk sac [194] Zebrafish embryo (morpholino *alk1*): Cerebral AVMs by 24 hpf, high-output heart failure by 3–4 dpf [196]	**Embryo (Alk1-Cre-L1, pulmonary EC): Lethal (E17.5); AVMs and dilated/tortuous vitelline arteries in E16.5 extraembryonic vasculature, and AVMs in E17.5 lung vasculature [239] **Neonate (Alk1-Cre-L1, pulmonary EC): Lethal (P5); Dilated, disorganized, tortuous blood vessels causing hemorrhage in brain/lung/small intestine. AVM shunts in brain and lungs [219] Neonate (Cdh5-CreERT2): Lethal ≤ 48 h following excision. Pulmonary hemorrhage, AVMs in pial vessels and GI tract [166, 168] Post-Natal Retina (Cdh5-CreERT2): Venous enlargement, vascular hyperbranching, ↑ vascular density, ↑ filopodia density, ↑ EC proliferation, AVMs, loss of arterial identity, ↓ pericyte coverage, ↓ pSMAD1/5/8 activity, ↓ endoglin expression [59, 166–168] Adult (Cdh5-CreERT2): Severe GI bleeding due to fragile microvessels in cecum villi, ↓ oxygen saturation due to hemorrhage, ↓ hematocrit and hemoglobin levels) [168] Adult (Scl-CreERT general EC): Lethal ~ 2 weeks following excision. AVM shunts (tortuous, enlarged vessels) in ear, GI tract, and skin wound areas. Severe cecal hemorrhage, fatal anemia. No effect on lymphatic vessels [227] Overexpression (Alk1-Cre-L1, Scl-CreERT, and RosaCreER): No pathological symptoms alone. Suppressed formation of AVMs in postnatal retinas and adult wounded skin in *Alk1*^*iECKO*^ and *Eng*^*iECKO*^ mice [192]
*Alk2*	Embryo: Lethal (< E9.5); Defects in mesoderm formation & gastrulation (abnormally thickened primitive streak, arrested development at late streak stage) [240, 241]. Arrested at early gastrulation stage, abnormal visceral endoderm morphology and severe disruption of mesoderm formation [242]	Embryo (Tie2-Cre): ↓ endocardial cushion size at E10.5, defects in heart septation and valve formation at E14.5, failure to undergo EndoMT [243] Post-Natal Retina (Cdh5-CreERT2): ↓ radial expansion and vascular density [59]
*Alk3*	Embryo: Lethal (< E9.5); No mesoderm formation, no gastrulation, thickened epiblast layer [244] Zebrafish embryo (morpholino, *alk3a/b*): ↓ Ephb4 expression, only one axial vessel present, lack of proper blood circulation [185] Adult (*Alk3*^+/−^): Normal, viable, fertile [244]	Embryo (Flk1-Cre): Lethal (E10.5–11.5); Defects in vessel remodeling and smooth muscle cell formation/recruitment, severe abdominal hemorrhage, AV canal endocardial cushion defects (↓ proliferation), anemic yolk sacs [226] Embryo (Tie1-Cre): Lethal (E11.5–12.5); Internal hemorrhage, ↓ mesenchymal AV cushion cells at E9.5–10.5, impaired EMT in AV canal [245] Embryo (Tie2-Cre): Lethal (E10.5); Severe growth retardation, lack of venous vessels, ↓ SMCs around dorsal aorta [185] Embryo (Dll4in3-Cre, arterial EC): No overt phenotypes [185] Post-Natal Retina (Cdh5-CreERT2): ↓ radial expansion, ↓ vascular density, ↓ sprouting at the angiogenic front [59]
*Alk6*	Embryo: Failure of metacarpals to segment, ↓ cell proliferation and ↑ cell death in digit regions [246] Neonate: Defects in appendicular skeleton (impaired chondrogenesis in proximal and middle phalanges region) [61] Adult: Viable; Defects in appendicular skeleton [61, 246]. Fertilization difficulty, irregular estrous cycle [247]	N/A
*Smad1/5/9*	Embryo (*Smad1*^*−/−*^): Lethal (E9.5); Impaired allantois formation, lack of placenta, disorganized vessels, lack of embryonic circulation [248, 249] Embryo (*Smad5*^*−/−*^): Lethal (E9.5–11.5); Impaired vasculogenesis and hematopoiesis, disorganized yolk sac vasculature, edema, hemorrhage in amnion, exteriorized heart, failure to close mid/hindgut and neural tubes [250]. Vascular development defects: enlarged blood vessels, fewer vascular smooth muscle cells, ↑ apoptosis of mesenchymal cells [251] Embryo (*Smad9*^*−/−*^): No overt defects; adults viable and fertile [252] Embryo (*Smad1*^+/−^*;Smad5*^+/−^): Lethal (E10.5); Impaired allantois morphogenesis, cardiac looping, and primordial germ cell specification [252] Zebrafish embryo (morpholino, *smad1/5*): ↓ *Ephb4* expression [185] Adult (*Smad9*^*LacZ−/−*^, aged 11.5 months): Lung vascular remodeling defects (media hyperplasia with vessel occlusion and plexiform lesions). A subset of mutants developed pulmonary adenomas. Heterozygotes: similar but milder phenotypes at a lower prevalence [253]	Embryo (*Smad1*^*f/f*^*; Smad5*^*f/f*^*;* Tie2-Cre): Lethal (E10.5); Severe vascular hemorrhage and edema, normal vasculogenesis but impaired angiogenesis, ↓ Dll4/Notch signaling, ↑ tip-cell-like cells (at expense of stalk cells) in E9.5 hindbrain and dorsal aorta but ↓ anastomoses of sprouts [228]. Spontaneous vascular shunt formation (AVM-like) between heart and dorsal aorta in yolk sacs at E9.25 [212] Post-Natal Retina (Cdh5-CreERT2): AVMs in high flow areas, ↓ functional tip cells at angiogenic front, ↑ vascular density in plexus, ↓ vessel regression, aberrant vascular loop formation [165]
*Smad4*	Embryo: Lethal (E6.5–8.5); Arrested growth before gastrulation due to ↓ cell proliferation, no mesoderm formation, abnormal visceral endoderm [254, 255] Embryo (Rosa-CreER): (Excised at E10.5) Disrupted arterial development, dilated coronary arteries, ↑ arterial EC size and proliferation. (Excised at E15.5) no change in vessel diameter [217] Neonate (Rosa-CreER): (Excised at P1) Lethal by P8, GI hemorrhage, dilated and tortuous AVMs in brain, intestine, nose, and retina [184] Post-Natal Retina (Rosa-CreER): (Excised at P1) AVM formation, aberrant smooth muscle actin staining, ↓ radial expansion [184] Adult (Rosa-CreER): Lethal ≤ 6 days of excision. ↓ weight and hemoglobin levels, GI hemorrhage, enlarged stomach/intestine/cecum, dilated and tortuous AVMs along GI tract and wounded skin [184]	Embryo (Flk1-Cre): Lethal (E9.5–10.5); ↓ hematopoietic colonies [226] Embryo (Tie2-Cre): Lethal (E9.5–10.5); Growth retardation, defects in vessel sprouting and remodeling, collapsed dorsal aortas, enlarged hearts with ↓ trabeculae, failed endocardial cushion formation, lack of *Ephb4* expression and absence of cardinal vein [185, 256] Embryo (Cdh5-CreERT2): (Excised at E9.5) Lethal (E13.5); Defective vein morphology, ↓ *Ephb4* expression [185] Embryo (Dll4in3-Cre, arterial EC): No overt phenotypes in embryos before E13.5, but lethal between E13.5 and P5 [185] Embryo (Apj-CreER, venous-derived EC): (Excised at E10.5) Dilated coronary arteries [217] Neonate (Cdh5-CreERT2): Lethal 4–8 days following excision. Defective lung vasculature and lung hemorrhage causing respiratory distress, AVMs in pial vessels and GI tract [166] Post-Natal Retina (Cdh5-CreERT2): AVM formation (in 82% of mutants), angiogenic defects, arteriovenous identity issues, ↑ artery/vein diameter, ↑ EC proliferation and size, altered mural cell coverage, ↓ Vegfr2 expression [183]. AVMs, ↑ vascular density and branchpoints at vascular front, ↑ EC proliferation in branching plexus, arteriovenous identity defects [166]
*Smad6*	Background-dependent, variable late-embryonic/perinatal lethality [257, 258] Embryo: Hemorrhage under skin [145]. Axial and appendicular skeletal defects [258] Neonate: Hyperplastic endocardial cushions, variable valve and outflow tract septation defects[257]. Domed skulls and short snouts [258] Post-Natal Retina: ↑ sprouting at the vascular front, ↑ density in branching plexus, disorganized EC junction markers [145] Adult: Ossification around outflow tracts of heart [257]	N/A
*Smad7*	Embryo: Significant postnatal lethality with cardiac defects: VSD, non-compaction; outflow tract (may result from elevated TFGb signaling) [259] Adult: small size, abnormal ECG, thin ventricular wall [259]	N/A
*Endoglin*	Embryo: Lethal (E10.0–11.5); Defective yolk sac vasculogenesis, embryonic angiogenesis, and vascular smooth muscle cell development; hemorrhage in yolk sac and embryo, cardiac malformations (enlarged ventricles and outflow tracts), cardiac cushion defects (failure to undergo EMT), delayed maturation of major vessels, severe anemia and ↓ red blood cell count [260–262] Adult (Rosa-CreER): ↓ weight and hemoglobin levels, GI hemorrhage, dilated and tortuous AVMs along GI tract and wounded skin [184]. AVM formation in brain following local VEGF stimulation [263] Adult (*Eng*^+/−^): Viable and fertile. Background-dependent and variable penetrance of telangiectases and dilated/tortuous vessels in skin, low frequency of AVMs [261, 262] Zebrafish embryo (mutant *eng*^*mu130*^): ↓ blood flow through ISVs, altered blood vessel diameters bypassing smaller ISVs to shunt through large arteries and veins, ↑ blood vessel pruning [220] Zebrafish adult (mutant e*ng*^*mu130*^): Survive to adulthood. Multiple vascular malformations, dilated/tortuous vessels in head, ↑ artery and vein diameter, but ↑ EC numbers in veins only [220]	Neonate (Cdh5-CreERT2): (Excised at P1) AVMs and ↑ tip cells in brain [169] Post-Natal Retina (Cdh5-CreERT2): Vascular hypersprouting, delayed capillary remodeling, severe AVM formation (20% of those were bleeding AVMs), αSMA expression no longer follows arteries specifically and is found on veins too, ↑ vessel branching at periphery, enlarged veins, ↑ EC proliferation [169, 170] Post-Natal Retina (Apj-CreERT2): AVMs in proximal and distal retina [264] Adult (Cdh5-CreERT2): ↓ angiogenesis and venomegaly (matrigel plug assay) [170] Adult (Scl-CreERT, general EC): Dilated/tortuous vessels and arteriovenous shunts in wounded skin [227] Adult (Sm22α-Cre): Lethal ~ 6 weeks of age. AVM formation in brain, spinal cord, and intestines, hemorrhage in some brain and spinal cord lesions [263]

*E10*.5 embryonic day 10.5, *P5* postnatal day 5, *LEC* lymphatic endothelial cells, *AVM* arteriovenous malformation, *DA* dorsal aorta, *CV* cardinal vein, *DORV* double-outlet right ventricle, *VSD* ventricular septal defect, *AV* atrioventricular, *PAH* pulmonary arterial hypertension, *RVSP* right ventricular systolic pressure, *VSMC/SMC* (vascular) smooth muscle cell, *hpf/dpf* hours (days) post-fertilization, *GI* gastrointestinal, *EC* endothelial cell, *EndoMT* endothelial-to-mesenchymal transition, *EMT* epithelial-to-mesenchymal transition

*Global mutant phenotypes may not be discussed in the text, but are included in Table 1 for comparison to endothelial-specific mutant phenotypes

**Divergent timing of similar phenotypes in Alk1f/f;Alk1-Cre-L1 mice attributed to different Cre-mediated recombination efficiencies

While the Type I receptors share general structures and downstream targets, small differences in their protein sequence contribute to differential ligand affinity. These receptors are most similar in the kinase domain and can be grouped by their homology (as measured by amino acid similarity) in this region: ALK1/ALK2 share 88%, and ALK3/ALK6 share 95% kinase domain amino acid homology, while all other Type 1 receptor pairings exhibit between 77 and 79% homology. Similarly, ALK1/ALK2 share the high homology in the GS domain (87%), while ALK3/ALK6 share only 54% GS domain homology. The BMP Type 1 receptors are the least homologous in the ligand-binding domain, with any two ALKs sharing merely 22–34% homology, although this divergence most often leads to binding preferences rather than rigorous ligand–receptor binding partners. The amino acid residues that differ in the ligand-binding domain of the ALKs create changes in the ligand-receptor interfaces that facilitate different stabilizing interactions [62].

Type I receptors provide significant selectivity for ligand binding in the heterotetrameric complex with Type II receptors [63–67]. Current thinking describes two major BMP signaling axes in endothelial cells that produce different phenotypic outputs: ALK1 binds BMP9 and BMP10 to signal homeostatic or anti-angiogenic BMP signaling, while ALK2 and ALK3 binding to BMP2, 4 and/or 6 facilitates proangiogenic signaling. Structural modeling reveals that the contact area between BMP ligands and Type 1 receptors is extensive and hydrophobic, although it lacks perfect surface complementarity, which may contribute to some promiscuity of interactions between ligands and receptors [68]. ALK1 was originally considered an orphan receptor or part of a complex with TGFβR2 to transduce TGFβ1 signals; however, it is now well characterized as a binding partner for BMP9 and BMP10, although it also binds TGFβ1 in endothelial cells [69, 70]. ALK1 interactions with BMP9/10 have remarkably high binding affinity (in the picomolar range) compared to the other BMP ligand/Type I receptor pairs that have affinities in the nanomolar range [71, 72]. ALK2 primarily binds BMP6, but it also binds BMP9 with lower affinity than BMP9-ALK1 binding [73, 74]. ALK3 is a demonstrated binding partner of BMP2, BMP4, and BMP6 [68, 75, 76].

### Endothelial cell BMP type II receptors

BMP Type II receptors are transmembrane serine/threonine kinases that efficiently phosphorylate Type I receptors once the heterotetrameric receptor–ligand complex is formed. They are structurally related to Type I receptors in that they include an N-terminal extracellular ligand-binding domain, a membrane-spanning α-helical domain, and a C-terminal cytoplasmic portion that includes the kinase domain [77] (Fig. 2C). However, Type II receptors also differ from Type I receptors in the cytoplasmic domain—they lack a GS domain and BMPR2 has a long carboxy-terminal tail [78]. Mutations in the BMPR2 tail disrupt SMAD-mediated signaling but activate non-canonical BMP pathways, such as p38, MAPK and ERK, in a ligand-independent manner. These non-canonical pathways are also activated by BMP4 ligand binding [79, 80]. The Type II receptor kinase domain is constitutively active; however, it is blocked from prematurely phosphorylating Type I receptors by the FK506-binding protein 12 (FKBP12). FKBP12 binds Type I receptor GS domains and blocks the active conformation, which is required for Type II receptor phosphorylation of the Type I receptor [81–83]. Release of FKBP12 from the Type I receptor requires an intact and functional Type II receptor kinase domain [84].

The BMP arm of the TGFβ superfamily has three Type II receptors, BMPR2, ACVR2A (ActRII) and ACVR2B (ActRIIB); of these, BMPR2 is thought to be most relevant to endothelial cell signaling, although ACVR2A functions to balance BMP and TGFβ signaling in pulmonary endothelial cells [85]. BMPR2 is the main Type II receptor involved in heterotetramers with ALK1 and ALK2 [86], and it is more highly expressed in adult mouse endothelial cells than the other BMP Type II receptors [54]. In mouse embryos, whole-mount in situ hybridization revealed that BMPR2 and ACVR2B had nearly ubiquitous expression in embryonic and extraembryonic tissues between E6.5 and 7.5, while ACVR2A expression was not detected [87]. BMPR2 expression was moderately widespread at E9.0–10.5, with highest expression levels in the AV canal, outflow tract, and limb buds and lower expression in the lungs; expression was not detected in the atria and ventricles of the heart [48].

### Endothelial cell BMP co-receptors

BMP co-receptors, also called Type III receptors, enhance the responses of receptor complexes to BMP ligands. Co-receptors have an extensive extracellular domain that interacts with and modulates the affinity of BMP ligands for Type I and II receptors, but they lack a substantial intracellular domain and do not signal on their own (Fig. 2D). Molecular and genetic data support that endoglin (ENG) functions in endothelial cells. Betaglycan (BG), which presents TGFβ to its Type II receptor, is also implicated in some aspects of endothelial BMP signaling via interactions with BMP2 and BMP4 to promote ligand binding to ALK3/6; thus, betaglycan may influence BMP signaling, although this has not been directly tested in endothelial cells [88]. These receptors share homology in transmembrane and cytoplasmic domains but diverge in the extracellular domains involved in receptor binding. Both receptors bind TGFβ1 and TGFβ3, while only betaglycan binds TGFβ2 [89, 90]. Betaglycan is co-expressed with ENG on human microvascular endothelial cells, where it binds TGFβ1-3 [91].

ENG strongly binds BMP9 and ALK1, potentiating BMP signaling through ALK1 [70, 73, 92, 93]. The interaction of ENG with BMP9-ALK1 signaling is especially important for flow-mediated responses of endothelial cells (see below). The BMP9 binding site for ENG overlaps its binding site for Type II receptors, implying that ENG does not remain in the receptor complex but is displaced once BMP9 binds the Type II receptor [94]. Structural studies indicate that ENG binds BMP9 in a manner analogous to an antibody binding an antigen: membrane-bound ENG is a dimer with two arms connected by a disulfide bridge that opens to engage BMP9 [95, 96] (Fig. 2D).

A soluble form of ENG (sENG) is produced in some situations. Matrix metalloproteinases can cleave the extracellular domain, producing sENG which downregulates pro-angiogenic proteins in human and mouse endothelial cells and inhibits angiogenesis, sprouting, and tube formation [94, 97, 98]. sENG has been proposed to act as a trap for BMP9 before it engages with surface ALK1 [94]. However, a recent study demonstrated sENG circulates as a monomer, and its binding with BMP9 does not inhibit BMP9 signaling, although it is most efficient in the presence of endogenous membrane-bound ENG [99]. Circulating sENG levels are elevated in pregnant women with pre-eclampsia [100], and ENG levels directly correlate with the severity of pre-eclampsia. Moreover, overexpression of sENG increases microvascular permeability in mice and promotes pre-eclampsia symptoms in pregnant rats [101], suggesting that circulating sENG contributes to pre-eclampsia. Paradoxically, anti-endoglin antibodies effectively block tumor angiogenesis while producing telangiectasias and vascular overgrowths as side effects [102–104]. Taken together, these findings suggest complex roles for ENG in angiogenesis and homeostatic barrier function in various contexts.

### BMP effectors in endothelial cells

After BMP ligands bind receptors on endothelial cells, canonical BMP signaling proceeds via phosphorylation of effector R-SMADs; phosphorylation changes R-SMAD conformation and allows for binding to the Co-SMAD, SMAD4. The R-SMADs involved in BMP signaling include SMAD1, 5, and 9 (SMAD9 was formerly referred to as SMAD8) [105]. The R-SMADs are highly homologous to each other, sharing over 96% amino acid similarity in their functional domains, and are thought to act redundantly in canonical BMP signaling (Fig. 2E). The R-SMADS and Co-SMAD share an N-terminal MH1 domain responsible for DNA binding, followed by a proline-rich linker region that connects to a C-terminal domain called MH2 (Fig. 2E, F). The MH2 domain mediates receptor recognition, nuclear import, and SMAD oligomerization.

SMAD4 is the common effector SMAD of both BMP and TGFβ signaling, and it transduces both proangiogenic and homeostatic BMP signaling in endothelial cells. The MH1 and MH2 domains of SMAD4 are highly homologous to those of the R-SMADs (Fig. 2E, F). The SMAD4 MH2 domain recognizes and binds the MH2 domain of two R-SMAD proteins to create a heterotrimer that enters the nucleus and binds DNA [106]. Bulk RNA sequencing of primary human endothelial cells found SMAD4 expressed in endothelial cells isolated from the aorta, coronary arteries, and umbilical artery and vein, and expression was higher in fresh than cultured cells [52, 53].

Canonical BMP signaling converges on signaling through the same R- and Co-SMADs, and despite their high homology, this signaling results in variable endothelial cell responses that are context-dependent, likely affected by the type and level of available BMP ligands, the expressed BMP receptors, and impacts from blood flow. For example, both the proangiogenic effects of BMP2 and the homeostatic effects of BMP9 increase nuclear pSMAD1/5/9 in endothelial cells, which is indicative of canonical BMP signaling. The mechanisms causing differences in endothelial cell signaling outcomes through these same R- and Co-SMADs remain to be elucidated.

### BMP antagonists and endothelial cells

BMP pathway antagonists are found both within endothelial cells and in the intercellular milieu. Inhibitory SMADs (i-SMADs: SMAD6 and SMAD7) are a class of SMADs that negatively regulate BMP signaling in a cell-intrinsic manner; SMAD6 is relatively selective for BMP signaling in its effects, while SMAD7 is thought to more broadly affect BMP/TGFβ signaling [19, 107]. Both i-SMADs are expressed in human endothelial cells, particularly in the aorta and freshly harvested umbilical endothelial cells [52, 53]. BMP signaling through SMAD1/5 upregulates expression of SMAD6 and SMAD7, which creates a negative feedback loop for the BMP signaling pathway [108]. SMAD6 expression is also upregulated by Notch signaling in endothelial cells [109, 110]. SMAD6 and SMAD7 can regulate BMP signaling in several ways. They contain a conserved MH2 domain that allows for competition with R-SMADs to bind Type I receptors or co-SMADs, thus blocking productive canonical BMP signaling (Fig. 2G) [111]. In non-endothelial cells, SMAD6 interacts strongly with ALK3 and ALK6 to inhibit BMP signaling while SMAD7 effectively blocks BMP signaling initiated by ALK1, ALK2, ALK3, and ALK6 [112]. SMAD6 also recruits SMURF1, an E3 ubiquitin ligase, to degrade R-SMADs and BMP Type I receptors [113]. SMURF1 regulates BMP signaling by directly binding SMAD1, SMAD5, and SMAD6 or SMAD7, as they all contain the PPXY target sequence that binds the WW2 domain on SMURF1 [114]. SMAD6 may also function in the nucleus, where it binds transcription factors like Homeobox (Hox) C-8 and prevents Hox–SMAD1 interactions [115, 116]. i-SMADs do not have a conserved MH1 domain, but their N-terminal domains determine subcellular localization and regulate nuclear export [117, 118]. SMAD6 and SMAD7 are also regulated via methylation on N-terminal arginine residues that influence their binding to BMP receptors and their ability to block BMP signaling [119, 120].

As with other aspects of BMP signaling, the context and concentration of an antagonist may alter outcomes. For example, BMPER is synthesized in endothelial cells and secreted into the extracellular matrix, where it binds BMP ligands to regulate their function. Its role as either agonist or antagonist of vascular BMP signaling is dose-dependent: at low concentrations, BMPER promoted sprouting and vessel formation, while at high concentrations it repressed these activities [121–123]. In vivo in mice and in vitro in human umbilical vein endothelial cells (HUVEC), reduced *Bmper* levels decreased endothelial cell barrier function, further cementing its role as an endothelial regulator [124].

Further fine-tuning of BMP signaling is achieved through extracellular antagonists, including Chordin, Noggin, Gremlin, and MGP (matrix gamma-carboxyglutamate protein). These proteins are secreted by non-endothelial cells in tissues, and in general they bind BMP ligands and prevent ligand access to BMP receptors (for a thorough review on BMP antagonist structure and binding to BMP ligands and receptors, see [125]). Chordin binds and inhibits BMP4, while Noggin and Gremlin bind and inhibit BMP2, BMP4, and BMP7 [36, 126–128]. Of note, a single lysine residue in BMP6 not present in BMP2 or BMP7 confers resistance to binding and inhibition by Noggin [129], and BMP9 and BMP10 are also resistant to Noggin inhibition [130]. MGP is an extracellular effector that binds BMP2 and BMP4 and is active in the vasculature [131, 132]. Like BMPER, MGP has biphasic effects, as it acts in a concentration-dependent manner as either an agonist or antagonist to BMP signaling in a feedback loop proposed to involve TGFβ activation of ALK1 [133].

## Chapter 2: BMP signaling in endothelial cell behaviors

Tissues cannot survive without a proper supply of oxygen and nutrients. The formation of a mature vascular network is complex and includes numerous processes, such as vasculogenesis, sprouting angiogenesis, branching, lumenization, remodeling and homeostasis [134, 135]. Vasculogenesis is the de novo formation of new blood vessels from precursor cells called angioblasts, and angiogenesis is the formation of new vessel conduits via sprouting and migration of endothelial cells from pre-existing vessels, with subsequent anastomosis (connection) and lumenization [136]. Remodeling involves loss of some conduits while others increase in diameter to eventually form a hierarchical vascular network that sets the final pattern of vessels. As organs mature and vascular remodeling diminishes, endothelial cells become homeostatic—they stop proliferating, align in the direction of blood flow, and set up a barrier that regulates blood-tissue exchange of oxygen and nutrients. These processes involve complex endothelial cell behaviors. For example, sprouting angiogenesis requires that endothelial cells perform a variety of acrobatics: activation of cell–cell adherens junctions, collective migration, proliferation, polarization in both proximal–distal and apical-basal axes, lumen formation, and extracellular matrix deposition. Homeostasis involves repression of the cell cycle (called quiescence), cytoskeletal rearrangements, and junction stabilization. Tight regulation of these cellular processes is important, and dysregulated blood vessel growth is often lethal during development, while ectopic overstimulated blood vessel growth is a hallmark of cancer and chronic inflammation [137–142]. Open questions include understanding how endothelial cells coordinate the numerous cellular behaviors involved in angiogenesis, and how sprouting endothelial cells transition from active sprouting to remodeling to a quiescent phenotype once sprouting angiogenesis is complete.

## Chapter 2.1: BMP and sprouting angiogenesis

BMP pathway members have been implicated in each of the steps required for new blood vessel formation. Numerous cell-based assays in two or three dimensions have assessed BMP signaling involvement in specific endothelial cell behaviors and overall sprouting. These assays recapitulate aspects of the complex in vivo expansion of vessel networks in experimentally tractable systems that are often better suited to identification of specific effects, and for unraveling mechanisms and epistatic relationships.

### Endothelial cell junction destabilization

**Fig. 3:**
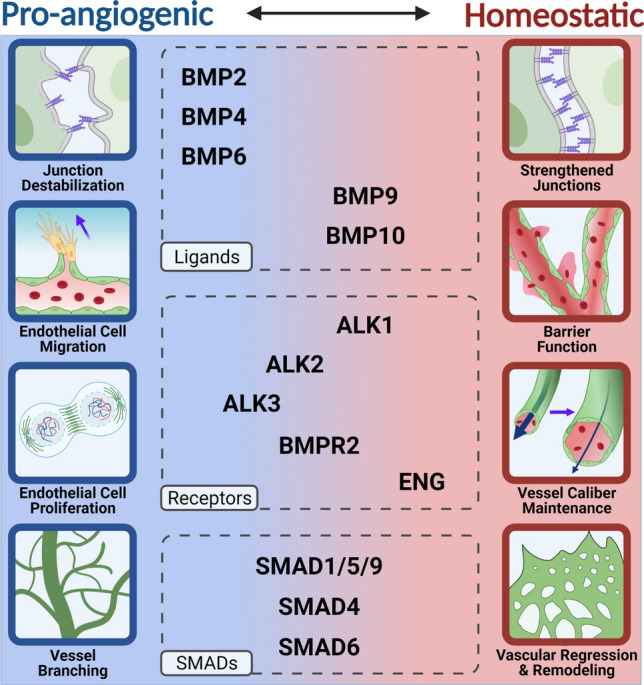
Proangiogenic vs. Homeostatic Activities of BMP Signaling. Pro-angiogenic activities of endothelial cells include junction destabilization, migration, proliferation, and vessel branching, while homeostatic activities include strengthened junctions, barrier function, maintenance of vessel caliber, and vascular pruning, regression, and remodeling. BMP2, BMP4, and BMP6 have clear pro-angiogenic effects while ENG, BMP9, BMP10, and ALK1 have mostly homeostatic effects on endothelial cells. However, there is overlap in the effects of the other endothelial BMP components, including ALK2, ALK3, BMPR2, SMAD1/5/8, SMAD4, and SMAD6 that indicate a mixture of pro-angiogenic and homeostatic contributions that may be context-dependent

A first step in sprouting angiogenesis involves dynamic rearrangement of endothelial cell–cell adherens junctions. Angiogenic sprouting proceeds by a form of collective migration, whereby endothelial cells maintain connections with other endothelial cells, but remodel junctions to change spatial relationships as sprouting proceeds [143]. Endothelial cell adherens junctions utilize VE-cadherin molecules on two different cells to form homophilic interactions at the cell membrane. Adherens junctions are modified through changes in the expression, phosphorylation, and internalization of VE-cadherin, and these changes alter the spatial relationship of endothelial cells to their neighbors as sprouts form, elongate and lumenize (for a thorough review of endothelial cell adherens junctions, see [144]). BMP ligands that are considered proangiogenic have destabilizing effects on adherens junctions (Fig. 3). For example, BMP6 enhanced sprouting in HUVEC in a 3D angiogenesis assay [109], and this sprouting was associated with phosphorylation and internalization of VE-cadherin [86, 145]. BMP4 also destabilized endothelial cell junctions: increasing BMP4 concentration decreased overall VE-cadherin protein expression in HUVEC and disrupted adherens junction patterning, while reducing BMP4 via siRNA knockdown increased VE-cadherin RNA expression [146]. BMP4 also affects leukocyte transmigration through an endothelial cell monolayer, a process important in inflammation; excess BMP4 stimulated transmigration while BMP4 knockdown had the opposite effect [146].

### Angiogenic sprouting

Tip cells are defined as the endothelial cells at the front of a sprout, and they guide sprout extension, usually by extending filopodia that sense the micro-environment [147]. Stalk cells are the endothelial cells found behind the tip cells, and they are characterized by collective migration behind the leading edge, higher proliferative capacity, and apical-basal polarization to form lumens [134, 148]. Nascent sprouts elongate through collective migration of polarized tip cells, and through proliferation of endothelial stalk cells, which trail behind the migratory tip cells [149, 150]. Tip cells are polarized in the proximal–distal axis, and they continuously rearrange with surrounding stalk cells in a dynamic competition; evidence for this exchange was seen ex vivo in aortic rings and in vitro in embryoid bodies, and there is evidence that this relationship holds in vivo in the postnatal retina [149, 151]. This dynamic behavior is regulated in part by VEGFA signaling that influences levels of Notch signaling [151]. The BMP signaling pathway is also important in the balance between tip and stalk cells in angiogenic sprouts. Notch signaling induced expression of the BMP inhibitor SMAD6 in endothelial cells to alter their responsiveness to proangiogenic BMP ligands, so that stalk cells had repressed responsiveness to BMP2 and BMP6 [109]. Another study also found that BMP2 and BMP6 increased overall sprouting in vitro, and this effect was linked to the Type I receptor ALK3, while ALK2 repressed sprout formation [57].

Endothelial cell behaviors linked to sprouting angiogenesis are often studied in vivo using the early postnatal retina [147], without the confounding phenotypes often found in the embryo proper. Although this vascular bed is one example of vascular sprouting and the phenotypes are likely to be context-dependent, it is amenable to rigorous quantification of endothelial cell behaviors and thus provides useful information. The vascular plexus initiates at birth via outward migration from the centrally located optic nerve in a planar manner, and during the first postnatal week the angiogenic front of this network is a spatially defined locale of new sprouting that can be measured [152]. In the postnatal retina, endothelial cell-selective genetic deletion of BMP pathway components reveals a proangiogenic role for signaling through *Alk2/3* and *Bmpr2*. *Alk2*, *Alk3* and *Bmpr2* enhance vascular density, and *Alk3* and *Bmpr2* augment endothelial cell sprouting at the vascular front, while *Alk1* prevents excessive vessel density at the front [59]. Gain-of-function experiments reveal that embryonic blood vessels respond to BMP signals with angiogenic responses. For example, induced overexpression of *bmp2b* in zebrafish embryos caused ectopic sprouting from the axial vein but not the dorsal aorta [55], and BMP4 overexpression along the notochord induced ectopic formation of a vascular plexus in a normally avascular area of the embryo [153]. However, the overall effects of most loss-of-function BMP pathway manipulations on sprouting angiogenesis in vivo are mild, and the physiological relevance of gain-of-function experiments is unclear.

How BMP9/10 and ALK1 affect angiogenic sprouting is complex. Early experiments involving the BMP9/ALK1 signaling axis demonstrated increased proliferation and migration of mouse endothelial cells upon stimulation with TGFβ or BMP9 or expression of constitutively active ALK1 [154, 155]. These results contrast with other reports that BMP9/ALK1 regulates vascular homeostasis by actively inhibiting endothelial cell migration, proliferation, and sprouting [59, 70, 73, 156–159]. Discrepancies in endothelial cell responses to BMP9/ALK1 signaling may result from different ligand concentrations, effects of unidentified serum-derived factors, or the presence/absence of flow forces. As with many aspects of BMP signaling, BMP9/ALK1 effects on endothelial cells are highly context-dependent, underscoring the importance of careful experimental design and analysis.

Many studies investigate proangiogenic BMP signaling in cultured primary endothelial cells. The phenotypes of genetic manipulations in animal models are complex, and they suggest overall that BMP signaling does not produce a strong unilateral proangiogenic signal in vivo, but rather a balance between proangiogenic and homeostatic BMP signaling that is important for formation and function of blood vessels. We posit that BMP signaling outputs differentially affect sprouting angiogenesis in vitro vs. in vivo because of a unique role for BMP signaling downstream of flow-mediated mechanotransduction in vascular endothelial cells. In this scenario, the outputs documented for proangiogenic signaling in vitro provide a picture of the *potential* of endothelial cells to respond to proangiogenic signals. This is important because pathological situations may tip the balance to a proangiogenic phenotype in vivo that is more than just the absence of a homeostatic signal—for example, BMP ligands are often elevated in tumors [160] and accompanied by excessive ectopic angiogenesis.

Why is BMP signaling not more prominent in vascular sprouting in vivo? This might reflect the fact that the components of proangiogenic BMP signaling are used iteratively during development in numerous tissues and organs. As discussed, BMP signaling is important for a wide variety of critical developmental processes, including embryonic patterning, lung morphogenesis, and bone formation. It is conceivable that the lack of specialization of this pathway, especially in comparison to pathways such as VEGFA whose expression and effects are much more tissue-restricted, makes it a difficult pathway to co-op purely for angiogenesis except in certain contexts. It might also result from the evolutionary addition of a BMP signaling module that functions in homeostasis in endothelial cells. Since embryonic vessels in vivo likely balance proangiogenic sprouting with homeostatic outputs, and BMP-induced homeostasis appears to be dominant under normal developmental conditions, this suggests that proangiogenic BMP signaling is not active developmentally in the vasculature, or that proangiogenic and homeostatic BMP signaling balance in complex ways in vivo.

## Chapter 2.2: BMP signaling in vascular patterning

### Vascular remodeling

Once a primitive vascular plexus forms via sprouting angiogenesis, flow-mediated signals lead to vessel remodeling. This involves the pruning of unnecessary conduits, regression of vessels no longer under flow, and expansion of other vessels to accommodate increased flow, sometimes with additional recruitment of smooth muscle cells and fibroblasts [161, 162]. In many vascular beds the endothelial cells in pruned vessels do not undergo cell death, but rather they migrate to vessels experiencing flow and contribute to their expansion [163, 164].

The increased vascular density induced by concurrent endothelial cell-selective deletion of both *Smad1* and *Smad5* in the postnatal retina was accompanied by reduced vessel regression [165]. Similarly, endothelial cell-selective deletion of *Smad4* in postnatal retinas increased vascular density and branchpoints, although this was attributed to increased endothelial cell proliferation [166]. Since impaired vascular regression is a hallmark of defective vascular remodeling, these findings suggest that *Smad4* and *Smad1/Smad5* function is important in remodeling to form a hierarchically branched vasculature. It should be noted that SMAD4 is common to both the TGFβ and BMP signaling pathways, while SMAD1/5 signals predominantly downstream of the BMP signaling arm, although the similarity in the deletion phenotypes suggests that BMP signaling regulates vascular remodeling in the early postnatal retina. Endothelial cell-selective deletion of *Alk1* in postnatal retinas also caused vascular hyper-branching and an increase in filopodial density, indicating that ALK1 is important for vascular pruning and regression [59, 167, 168]. Endothelial cell-selective deletion of *Eng*, a co-receptor of ALK1 in transducing endothelial cell flow-mediated responses, caused a similar hyper-proliferative vascular phenotype in retinas: veins were enlarged due to increased endothelial cell proliferation and peripheral vessel branching was increased [169, 170]. Taken together, these studies indicate that BMP signaling affects vascular remodeling during developmental angiogenesis in complex ways.

Vessel remodeling also occurs in pathologies. For example, Pulmonary Arterial Hypertension (PAH) results in abnormal vascular remodeling and hypertension and is linked to *BMPR2* mutations in patients, and mice lacking one copy of *Bmpr2* had mild pulmonary hypertension and impaired pulmonary vascular remodeling under hypoxic conditions, a phenotype reminiscent of human PAH [171]. Moreover, embryonic deletion of *Bmpr2* using an ALK1-L1-Cre driver that is expressed in E9.5 extraembryonic vessels and is predominant in E13.5 lung endothelial cells, resulted in a predisposition for adult mice to develop PAH [172, 173]. BMP9 is also implicated in PAH, via both mutations (*GDF2*) associated with PAH patients and animal studies [174, 175]. These studies implicate BMP signaling in pulmonary vascular remodeling, although the underlying mechanisms are not well understood.

### Arteriovenous identity

Arteries and veins have different functions, and they maintain unique identities to form a circuit delivering oxygenated blood to peripheral tissues and returning deoxygenated blood to the heart. Differences between arteries and veins include vessel-specific gene expression, the composition and thickness of surrounding mural cells and extracellular matrix, and vessel diameter (for detailed reviews see [176–179]). Hemodynamic forces reinforce arteriovenous differentiation, which during development is genetically initiated prior to the onset of blood flow. This is exemplified in live imaging of chick embryo yolk sacs, where expression of arterial markers was flow-regulated [180], and in mouse embryos where Notch1 signaling, an important factor in determining arteriovenous identity, became elevated and localized to arteries with the onset of flow [181]. Although the initial artery–vein determination in early development closely coincides with endothelial cell differentiation and may involve BMP signaling in complex ways, BMP is also important in the subsequent flow-regulated maintenance of artery–vein identity. For example, postnatal deletion of *Eng* in endothelial cells led to ectopic expression of the smooth muscle marker αSMA on retinal veins, consistent with loss of arteriovenous identity [169]. Notch signaling also rescued loss of the arterial marker EphrinB2 downstream of ALK1 loss in endothelial cells [182]. Postnatal endothelial cell deletion of *Smad4* also led to arteriovenous identity perturbations in retinal vessels, while earlier deletion caused dysfunctional venous structures with reduced venous marker expression in embryos [166, 183–185]. Endothelial cell-selective deletion of *Alk3* developmentally resulted in stunted embryos that made arteries but not veins; the early lethality suggests that the deletion affects primary artery–vein identity rather than flow-mediated maintenance of artery–vein identity [185].

### Arteriovenous malformations/HHT

Arteriovenous malformations (AVMs) are aberrant connections between arteries and veins that shunt blood directly between the large vessels, bypassing normal capillary beds. The exact mechanisms of AVM formation are still unknown, but AVMs are associated with dysregulation of all the functions discussed above: vascular remodeling, vessel caliber, endothelial barrier function, and arteriovenous identity. Evidence supports a multi-hit model of AVM formation that involves both genetic and environmental changes. First, heterozygosity for various BMP pathway components is followed by mosaic loss-of-heterozygosity to generate patches of mutant endothelial cells in the vasculature [186]. Along with the genetic lesion in a predisposed (heterozygous) background, an environmental disturbance that causes increased angiogenesis and/or inflammation in the context of altered shear stress and blood flow patterns is necessary to generate an AVM [187]. Mutations in *ENG, ALK1*, *SMAD4 and GDF2 (Bmp9)* are linked to human diseases involving AVM formation [9, 11, 27, 188, 189]. Such diseases include Hereditary Hemorrhagic Telangiectasia (HHT), where the resulting AVMs are prone to vessel wall fragility; this fragility leads to hemorrhage, which can be fatal if located in the brain, liver, or lungs [190].

AVM formation is a hallmark of mutations in BMP pathway genes in mice as well, although not all BMP mutations lead to AVMs, and AVM formation is not exclusive to BMP pathway mutations (i.e., Notch) (see Table 1 and [190]). Endothelial cell-selective *Eng* deletion in postnatal retinas led to 70% of animals developing AVMs, and 20% of these AVMs led to significant bleeding [169]. Moreover, in a neonatal retina model of HHT1 in mice lacking endogenous ENG, sENG treatment reduces AVM incidence, which indicates that sENG may enhance BMP9/10 signaling in the absence of membrane-bound ENG [97]. Loss of *Alk1* either globally in embryos or postnatally in endothelial cells led to AVM formation, and in retinas the AVMs formed only in areas of high blood flow [166–168, 191]. Over-expression of *Alk1* in adult mice did not obviously perturb the vasculature but prevented AVM formation caused by endothelial cell loss of *Alk1* in retinas and wounded skin [192]. In zebrafish, mutant *alk1* loss-of-function embryos developed dilated high-flow cranial AVMs [193–195]*.* Additionally, *alk1* disruption via morpholino knockdown led to cerebral AVM formation and dilated cranial vessels [196]. Neonatal *Smad4* global knockout mice developed AVMs in the brain, intestine, nose, and retinas, while adults formed AVMs in the gastrointestinal tract and skin following wounding [184]. Postnatal endothelial cell-selective *Smad4* deletion led to AVM formation in retinal vessels [166, 183]. Taken together, these data suggest that vascular AVMs are a hallmark of several BMP pathway manipulations in endothelial cells of mice and fish, including *Alk1*, *Eng*, and *Smad4*, all genes that are linked to AVMs in humans.

## Chapter 2.3: BMP signaling and the transition to vascular homeostasis

Once vessels mature, angiogenic sprouting is inhibited and homeostasis is actively maintained to sustain barrier function and preserve arteriovenous identity and vessel caliber [197, 198]. Dysregulation of vascular homeostasis is primary to numerous conditions, such as atherosclerosis and aortic aneurysms, and it is also secondary in diseases such as cancer, where blood vessels exhibit excessive and unregulated growth to feed tumors and bypass normal vascular beds. Tumor vessels possess abnormal physiology, and are often tortuous, leaky, and have ambiguous arteriovenous identity [199]. Here we focus on how BMP signaling functions as blood vessels transition from active growth and remodeling to a homeostatic or quiescent state. We are beginning to understand the inputs that affect this transition, although an understanding of how canonical BMP signaling is interpreted by endothelial cells to produce proangiogenic vs. homeostatic signals remains unclear.

### Blood flow-induced responses

The transition from active vessel sprouting to homeostasis is largely initiated by the onset of blood flow through new conduits formed via anastomosis and lumenogenesis [200, 201]. Movement of blood through lumenized vessels produces distinct physical forces on the endothelial cell lining [202]. Specifically, endothelial cell behaviors in response to fluid shear stress have been extensively examined, and a large body of published work in vitro and in vivo highlights the effects of fluid shear stress on endothelial cell morphology, gene expression and signaling. Endothelial cells receive, interpret, and respond to mechanical flow forces through various mechanosensory complexes. These mechanosensory complexes allow endothelial cells to translate mechanical signals into biochemical outputs that alter cell behavior, cell shape, and gene expression [203]. One well-characterized complex is composed of VE-cadherin, PECAM and VEGFR2 at the cell membrane, while another direct sensor is thought to be Notch1 [204–206]. Excellent recent reviews document the effects of mechanical forces on endothelial cells and describe different mechanotransduction mechanisms (see [207, 208]). Here we focus on how BMP signaling, likely downstream of the initial mechanical to biochemical signal transition, modulates endothelial cell responses to laminar shear stress signals to promote vascular homeostasis.

Vascular BMP signaling is crucial for blood vessel homeostasis downstream of mechanical forces. Fluid shear stress significantly affects the expression of several BMP signaling components including ALK1, SMAD1/5, SMAD6 and SMAD7 [110, 195, 209, 210]. Laminar flow-mediated BMP signaling also requires SMAD4 and leads to elevated levels of phosphorylated R-SMAD1/5 in the nucleus and regulation of several BMP target genes [211]. In vitro, endothelial cell responsiveness to BMP9 was increased by laminar flow, and both ALK1 and ENG were required for this flow-mediated change in sensitivity, while BMP9-mediated signaling absent flow required ALK1, but not ENG [167]. Further in vitro studies found that SMAD1/5/9 is phosphorylated in a BMP9-dependent manner in endothelial cells under laminar flow [212]. Endothelial cells also respond to different levels of shear stress, and BMP signaling is involved in these differential responses [213]. For example, the lower shear stress associated with veins allows primary cilia to persist, and these structures may sensitize endothelial cells to BMP9 signaling [214].

While it is not well understood how and where BMP signaling receives the initial signals from the primary mechanotransduction pathways, there are several candidates for integration points. For example, Notch is thought to be a direct mechanosensor, and Notch and BMP signaling intersect in the nucleus, where complexes of NICD and SMAD4 bind target gene promoters and affect gene expression [211, 215]. Notch and BMP signaling also integrate via SMAD6, a negative effector of BMP signaling. Notch upregulated SMAD6 expression, Notch signaling reduced BMP6-stimulated canonical BMP signaling in a SMAD6-dependent manner, and SMAD6 rescued the blunted flow alignment of endothelial cells under laminar flow due to loss of Notch signaling [109, 110]. These findings indicate that Notch transduces mechanosensory signals in part via interactions with BMP pathway components.

Downstream of initial endothelial cell responses to flow, laminar flow-induced SMAD1/5 activation is implicated in regulating aspects of the endothelial cell cycle. Oscillatory shear stress in human endothelial cells led to sustained phosphorylation and activation of SMAD1/5 and continued cell cycle progression independent of BMP2 or BMP4 [209]. Conversely, endothelial cells slowed the cell cycle as they became quiescent in response to laminar shear stress [110, 216]. These findings indicate that the type of flow-induced stimulus influences the ultimate endothelial cell response to canonical BMP signaling changes.

### Maintenance of vessel caliber

Flow-induced forces are coordinated with alterations of vessel diameter, or caliber, to ensure that overall forces remain homeostatic. BMP signaling influences blood vessel size through regulation of both endothelial cell proliferation and cell shape changes. For example, SMAD4 is crucial for regulating vessel diameter during vascular development, since postnatal endothelial cell-selective *Smad4* deletion increased endothelial cell proliferation and increased both artery and vein diameters in retinal vessels [183]. Embryonic *Smad4* deletion at E10.5 in coronary vasculature caused dilated coronary arteries through increased endothelial cell size and proliferation [217]. Interestingly, the latter phenotype was not seen when *Smad4* was deleted at E15.5 using the same driver, suggesting that BMP signaling regulates vessel caliber during development in a stage-specific manner.

Global loss of *Alk1* caused hyper-dilation of large vessels at E9.5 in mouse embryos [218] and when *Alk1* was globally deleted in adulthood using a ubiquitous inducible Cre driver [184, 219]. Zebrafish embryos with loss-of-function mutation for *alk1* had increased endothelial cell numbers in cranial vessels, leading to enlargement of high-flow mid- and hindbrain vessels [193]. Zebrafish embryos and adults with a loss-of-function mutation for *eng* had enlarged blood vessel diameters with increased blood vessel pruning but increased cell size, and in some cases increased cell numbers accompanied the increase in size [220]. Taken together, these studies indicate that BMP signaling through ALK1 and ENG regulate vessel caliber, and they suggest that flow responses mediated through this arm of BMP signaling are important in this regulation.

### Endothelial cell barrier function

Although endothelial cell adherens junctions must be activated to initiate sprouting angiogenesis and remodeling, their stabilization is critical once a new conduit has formed, and blood vessels establish a barrier that regulates the movement of fluid and small molecules into tissues and prevents leak. Several lines of evidence indicate that BMP signaling is crucial for maintenance of barrier function. The Type II receptor BMPR2 is required for barrier function of pulmonary endothelial cells in vitro, as reducing BMPR2 levels via knockdown increased albumin leak in a permeability assay [173]. Supporting these data, siRNA knockdown of BMPR2 in primary endothelial cells promoted inflammation as measured by a monocyte adhesion assay; this finding was supported in vivo*,* as *Bmpr2*^±^*;ApoE*^*−/−*^ mice had increased expression of inflammatory markers ICAM-1 and VCAM-1 in arterial endothelium [221]. BMP9-mediated signaling through Type I receptor ALK1 stabilized endothelial cell barrier function by preventing VE-cadherin phosphorylation and internalization, and by inducing expression of occludin, a tight junction protein, in a hyperglycemic environment [222]. BMPR2 and ALK2 were shown to physically interact with VE-cadherin in endothelial cells using a proximity ligation assay and immunoprecipitation [86]. Additionally, VE-cadherin also co-immunoprecipitated with ALK1 and ENG, providing further support for the intertwined activity of BMP signaling and adherens junction function [223].

Paradoxically, while some positive effectors of BMP signaling contribute to junction stabilization and vessel homeostasis, suppression of BMP signaling through various negative regulators also stabilizes endothelial cell adherens junctions. SMAD6 stabilizes endothelial cell adherens junctions and blocks leak, as reduced SMAD6 expression increased VE-cadherin turnover, disrupted junction patterning, and reduced barrier function in vitro [110, 145]. Additionally, suppression of BMP signaling via BMPER restored endothelial cell barrier function perturbed by addition of BMP4 in HUVEC transwell assays [124]. Heterozygous *Bmper*^±^ mice had increased vascular leak as demonstrated by increased dye egress in the lungs [124]. Transcriptional co-activators YAP and TAZ, best known as effectors of the Hippo signaling pathway, strongly inhibited BMP signaling both in vitro and in vivo to regulate adherens junction morphology and stability [224]. Reduced expression of YAP/TAZ in endothelial cells increased monolayer permeability to dextran, while loss-of-function mice exhibited increased dye leak in early postnatal brains [225]. The ability of both positive and negative BMP signaling to promote endothelial barrier function emphasizes the complexity of this pathway, and the importance of considering context in interpreting the effects of BMP pathway manipulations.

### Hemorrhage

Hemorrhage occurs when blood vessels lose integrity or are not connected in their proper hierarchical pattern, leading to loss of blood to the interstitial space. Hemorrhage is a common outcome of loss-of-function mutations in BMP pathway components in endothelial cells in vivo, and hemorrhage is often observed in multiple tissues across different time points. For example, excision of *Alk3* in endothelial cells using an Flk1-Cre driver caused severe abdominal hemorrhage in embryos at E10.5 [226]. Neonatal mice lacking *Alk1* exhibited pulmonary hemorrhage while adults had cecal hemorrhage [166, 168, 227]. Global deletion of *Smad4* in neonates or adults led to bleeding in the gastrointestinal tract [184]. Compound endothelial cell-selective loss of both *Smad1* and *Smad5* caused severe generalized hemorrhage [228]. Similarly, global deletion of *Smad6* also led to embryonic hemorrhage [145]. The cause of hemorrhage in these mutants has not been extensively analyzed, and there may be different upstream perturbations that result in the broad characterization of “hemorrhage”. However, *Smad6* mutant embryos had evidence of destabilized adherens junctions, which is associated with vascular fragility [145]. Further work characterizing effects of other BMP pathway manipulations on adherens junctions should further our understanding of this complex relationship.

## Chapter 3: BMP and blood vessels: key questions remaining

BMP signaling influences endothelial cell behaviors to regulate blood vessel formation and vascular homeostasis, and BMP signaling dysregulation directly leads to or is found downstream of significant vascular diseases. Despite a substantial body of cutting-edge research over the last decade, our understanding of how BMP signaling regulates endothelial cell behaviors involved in vascular function is incomplete, and numerous important questions remain. Many open questions derive from the context-dependence of the outcomes of BMP signaling in endothelial cells. This context-dependence can be simplified to a proangiogenic signaling arm and an anti-angiogenic or homeostatic signaling arm for BMP signaling in endothelial cells, although there is likely much more complexity to these processes, and these complex inter-pathway interactions are an area of active investigation.

One important question centers around how endothelial cells “read” BMP signaling and translate this signaling into cellular behaviors. Canonical BMP signaling results in phosphorylation of R-SMADs and their translocation to the nucleus, chaperoned by SMAD4, where they affect gene transcription, and both signaling arms stimulate nuclear translocation of pSMAD1/5/9. Target genes, such as *Id1*, *Smad6*, and *Apelin*, seem similarly regulated by both pro- and anti-angiogenic BMP ligands [211], although a recent paper examining transcriptional changes upon expression of constitutively active Type I receptors in endothelial cells revealed both shared and unique transcriptional targets, supporting the idea that BMP Type I receptors regulate different phenotypic responses [229]. Despite the relative similarity of downstream expression changes that have been interrogated, endothelial cell responses not only differ downstream of activation of Alk1 (homeostatic) vs. Alk2/3 (proangiogenic) receptor complexes, but in some cases, the resulting phenotypes are polar opposites. Thus, one or more aspects of BMP signaling must be differentially experienced by endothelial cells to provide context to the different types of signals. Based on evidence from other signaling pathways and from computational modeling of the BMP pathway, endothelial cells may respond to different signal amplitudes, signal durations, signaling location within the endothelial cell, different co-signaling contexts, or other aspects of signaling output. Modeling based on outputs in other cell types [230] suggests that some signals are sampled over a short time span to provide an “analog” output based on amplitude, while other signals are temporally sampled and the outputs integrated over time to provide the signal. It will be interesting to perform signaling experiments that address analog vs. integrated outputs in endothelial cells to determine cell type-specific BMP signal attributes. However, even this approach has limitations because the anti-angiogenic (homeostatic) arm of BMP signaling is flow-responsive, so further careful signaling experiments will be needed under different flow conditions to clearly delineate how endothelial cells distinguish BMP signaling inputs and respond with proangiogenic vs. homeostatic endothelial cell behaviors.

A second related question with strong ramifications for treating vascular diseases with a BMP component is—how do the two arms of BMP signaling integrate and balance each other in endothelial cells to produce the requisite behaviors? In a previous review, we described a simple model suggesting that homeostatic signaling is dominant over proangiogenic signaling once vessels establish blood flow relatively early in development [231]. In this conception, endothelial cell behaviors respond to the dominant signaling axis that uses BMP9/10 to signal through receptor complexes containing ALK1 and ENG, and proangiogenic signaling via other BMP ligands, such as BMP2/4/6 and receptor complexes containing ALK2 or ALK3, become irrelevant for endothelial cell behaviors. However, several lines of evidence suggest that this model is over-simplified. First, removal of components of the homeostatic arm, such as ALK1 or ENG, does not uncover a proangiogenic phenotype in most cases, as predicted by the model, but rather results in complex behavioral changes leading to hemorrhage and AVMs. Second, a loss-of-function mutation for *Smad4*, which is common to both pathways, phenocopies loss of the homeostatic arm via ALK1 or ENG but does not reveal a SMAD4-dependent proangiogenic phenotype that is also compromised. Finally, a negative regulator of proangiogenic BMP signaling, SMAD6, is functionally required for endothelial cell flow alignment [110], a finding that does not easily fit a model where proangiogenic signaling is irrelevant for homeostatic BMP signaling.

A third question centers around how BMP signaling is transduced mechanistically within endothelial cells to give rise to the relevant cellular behaviors. While there is some evidence that BMP receptor complexes may physically associate with endothelial cell junction components downstream of ligand engagement, how these interactions lead to changes in cell–cell junctions is not well-elucidated. Moreover, many BMP signaling effects on endothelial cell behaviors, such as proliferation, migration, and junction stability, are mediated via SMAD-dependent canonical BMP signaling that goes through the nucleus to affect gene transcription, and how canonical BMP-induced transcriptional changes lead to changes in cell behaviors is not well understood. A better understanding of these relationships will likely be helped by the recent surge in transcriptomic data, and especially from single-cell RNA seq data that generate endothelial cell transcriptomes derived from animal tissues and organs. This in vivo endothelial cell profiling is predicted to show effects of BMP manipulations in a relevant micro-environmental context. Additional questions center around indirect effects of BMP signaling on endothelial cell behaviors. For example, BMP signaling affects arteriovenous identity in complex ways that are poorly understood, so perhaps endothelial cell behaviors associated with these identities, such as cell cycle progression, are altered by BMP manipulations downstream of initial identity. In that regard, reduced levels of SMAD6 that blunt morphological responses to laminar flow also prevented flow-mediated quiescence and kept endothelial cells in the cell cycle [110].

Finally, it will be exciting to determine how the unique features of BMP signaling that affect endothelial cell behaviors can be harnessed to develop interventions that help in diseases caused by perturbations in vascular BMP signaling, such as HHT, PAH, and in diseases in which perturbed BMP signaling may be downstream of the initial lesion, such as CCM (cerebral cavernous malformations). For example, FK506 is a compound that prevents FKBP12 inhibition of BMPR2 signaling in blood vessels, and the restoration of BMP signaling afforded by the drug ameliorates symptoms in mouse models of PAH [83]. As our knowledge of the complex and fascinating roles of BMP signaling on endothelial cell behaviors and blood vessel formation and function are further understood, other therapeutic targets will likely be identified.

## Data Availability

Not applicable.

## Abbreviations

BMP: Bone morphogenetic protein
VEGF: Vascular endothelial growth factor
TGFβ: Transforming growth factor β
HHT: Hereditary hemorrhagic telangiectasia
ALK: Activin-like kinase
ENG: Endoglin
AVM: Arteriovenous malformation
AV: Atrioventricular
EndoMT: Endothelial-to-mesenchymal transition
E10.5: Embryonic day 10.5
P5: Postnatal day 5
R-SMAD: Receptor-mediated SMAD
Co-SMAD: Common SMAD
i-SMAD: Inhibitory SMAD
PAH: Pulmonary arterial hypertension
MGP: Matrix Gla protein
BMPER: BMP endothelial cell precursor-derived regulator
HUVEC: Human umbilical vein endothelial cells
